# Few-Body Bound States and Resonances in Finite Volume

**DOI:** 10.1007/s00601-020-01550-8

**Published:** 2020-06-29

**Authors:** Sebastian König

**Affiliations:** 1 Department of Physics, Technische Universität Darmstadt, 64289 Darmstadt, Germany; 2 ExtreMe Matter Institute EMMI, GSI Helmholtzzentrum für Schwerionenforschung GmbH, 64291 Darmstadt, Germany; 3 Department of Physics, North Carolina State University, Raleigh, NC 27695 USA

## Abstract

Since the pioneering work of Lüscher in the 1980s it is well known that considering quantum systems in finite volume, specifically, finite periodic boxes, can be used as a powerful computational tool to extract physical observables. While this formalism has been worked out in great detail in the two-body sector, much effort is currently being invested into deriving analogous relations for systems with more constituents. This work is relevant not only for nuclear physics, where lattice methods are now able to calculate few- and many-nucleon states, but also for other fields such as simulations of cold atoms. This article discusses recent progress regarding the extraction of few-body bound-state and resonance properties from finite-volume calculations of systems with an arbitrary number of constituents.

## Introduction

It is well known from the pioneering work of Lüscher [1–3] that simulating physical systems in a finite volume can be used as a tool to extract physical properties. The bound-state relation connects the finite-volume correction of binding energies to the asymptotic properties of the two-particle wavefunction, whereas for elastic scattering physical scattering parameters are encoded in the volume dependence of discrete energy levels. Resonances, i.e., short-lived, unstable states, are manifest in this discrete spectrum as avoided crossing of energy levels as the size of the volume is varied [4–6].

All this work is based on the fact that the physical S-matrix governs the volume dependence of energy levels and is widely used in Lattice QCD (LQCD). It has been extended in several directions, including non-zero angular momenta [7–9], moving frames [6, 10–13], generalized boundary conditions [14–18], particles with intrinsic spin [19], and perturbative Coulomb corrections [20].

To date, most results have been obtained for two-body systems. As numerical techniques, such as LQCD and in particular lattice effective field theory (LEFT) [21–23], progress to calculate states with an increasing number of constituents, understanding the volume dependence of more complex systems is of great relevance. This is particularly true for the study of few-body resonances in light of recent efforts to observe [24] and calculate [25–33] few-neutron resonances in nuclear physics.

Early studies of the triton and Efimov trimers in finite volume [34–37] derived explicit results for these bound systems. Generally, however, finite-volume three-body systems have a complicated structure [38], the understanding of which is an area of very active current research [39–48].

This article goes in a more general direction and considers what can be said about the volume dependence of systems with an arbitrary number *N* of constituents. Summarizing (and elaborating on) original work presented in Refs. [49, 50], a general overview of the *N*-body setup in Sect. 1.1 is followed by a discussion of bound states and resonances in Sects. 2 and 3, respectively. For bound states, the emphasis is on formal developments, while the finite-volume study of few-body resonances is more exploratory to date and the focus is therefore on an efficient numerical framework to look for such states. A conclusion and outlook to future work is provided in Sect. 4.

### General Setup

Let $|\psi \rangle$ describe a nonrelativistic quantum state of *N* particles in *d* spatial dimensions with masses $m_1,\cdots m_N$, using units where $\hbar =c=1$. The position-space wavefunction of this state can be written as $\psi ({\mathbf {r}}_1,\cdots {\mathbf {r}}_N)$, where ${\mathbf {r}}_i$ labels the coordinate of the *i*th particle in the system. $|\psi \rangle$ is assumed to be an eigenstate of a Hamiltonian

1 $$\begin{aligned} {\hat{H}}_{1\cdots N} = \sum _{i=1}^N {\hat{K}}_{i}+{\hat{V}}_{1\cdots N} \,, \end{aligned}$$

where ${\hat{K}}_{i} = {-\varvec{\nabla }}^2_i/{(2m_i)}$ and the potential term ${\hat{V}}_{1\cdots N}$ includes in general nonlocal interactions of every kind from two-particle up to *N*-particle interactions:2where3conveniently accounts for spectator particles and the $W_{i,\cdots }$ are integral kernels involving an increasing number of coordinates as one goes from two-body towards three- and higher-body interactions.

Different kinds of relative coordinates will be used in the following. For numerical calculations it is most convenient (for the implementations discussed in this work) to work with simple relative coordinates defined as

4 $$\begin{aligned} \mathbf {x}_i = \sum \limits _{j=1}^N U_{ij} \mathbf {r}_j \,, \end{aligned}$$

where

5 $$\begin{aligned} U_{ij} = {\left\{ \begin{array}{ll} \delta _{ij}\,, &{}\text {for}\quad i,j< N\,, \\ {-1}\,, &{}\text {for}\quad i < N,\, j = N\,, \\ 1/N\,, &{}\text {for}\quad i = N\,. \end{array}\right. } \end{aligned}$$

That is, all particle coordinates are expressed relative to the last particle. Note that for $i=N$ this definition includes the overall center-of-mass (c.m.) coordinate.

Assuming the interactions to respect Galilean invariance, the c.m. momentum is conserved and the c.m. kinetic energy decouples from the relative motion of the *N*-particle system. The kernels $W_{i,\cdots }$ can be expressed in terms of the $\mathbf {x}_i$, and by rotational symmetry they depend only on absolute values of pairwise relative distances. For the special case of local interactions one has

6 $$\begin{aligned} W_{i,j,\cdots }({\mathbf {r}}_i,{\mathbf {r}}_j,\cdots ;{\mathbf {r}}'_i,{\mathbf {r}}'_j,\cdots ) = V_{i,j,\cdots }\big (\{\left| \mathbf {x}_i\right| ,\left| \mathbf {x}_{i}-\mathbf {x}_{j}\right| _{i< j}\}\big ) \prod _{k} \delta ^{(d)}({\mathbf {x}}'_k-{\mathbf {x}}_k) \,. \end{aligned}$$

The notation for the arguments on the right-hand side is meant to indicate that the $V_{i,j,\cdots }$ are functions of some subset of $\left| \mathbf {x}_i\right|$ and $\left| \mathbf {x}_{i}-\mathbf {x}_{j}\right|$, which is sufficient to recover the relative distances between all interacting particle pairs. For a two-body system, the expression reduces to the familiar $V_{1,2}(\left| \mathbf {x}_1\right| )\,\delta ^{(d)}({\mathbf {x}}'_1-{\mathbf {x}}_1)$.

Throughout the rest of this work it is assumed that every interaction has finite range, i.e., each $W_{i_1\cdots i_k}$ vanishes whenever the separation between any pair of incoming or outgoing coordinates exceeds some finite length. The overall range *R* of ${\hat{V}}_{1\cdots N}$ is defined as the maximum of all the individual finite ranges.

## Bound States

Consider now an *N*-particle bound state with total c.m. momentum zero, energy $E={-}B_N<0$, and wave function $\psi ^B_N({\mathbf {r}}_1,\cdots {\mathbf {r}}_N)$. The finite-volume behavior of this state, that is, the functional form of the volume-dependent binding energy $B_N(L)$, is linked to the asymptotic properties of the wavefunction when one of the coordinates becomes asymptotically large while keeping the others fixed. Without loss of generality, it suffices to consider the limit $\left| \mathbf {r}_1\right| \rightarrow \infty$.

Let $\mathcal {S}$ denote the set of coordinate points $\{\mathbf {r}_1,\cdots \mathbf {r}_N\}$ where $\mathbf {r}_1$ is separated by a distance greater than *R* from all other coordinates so that within $\mathcal {S}$ there are no interactions coupling $\mathbf {r}_1$ to $\mathbf {r}_2,\cdots \mathbf {r}_N$. By the assumption of vanishing c.m. momentum, it suffices to consider the reduced Hamiltonian

7 $$\begin{aligned} \sum _{i=2}^{N} {\hat{K}}_i - {\hat{K}}^{\text {CM}}_{2\cdots N} + {\hat{V}}_{2\cdots N} + {\hat{K}}^{\text {rel}}_{1|N-1} \,, \end{aligned}$$

where $${\hat{K}}^{\text {CM}}_{2\cdots N} = {-}({\varvec{\nabla }}_2+\cdots {\varvec{\nabla }}_{N})^2/(2m_{2 \cdots N})$$ and

8 $$\begin{aligned} {\hat{K}}^{\text {rel}}_{1|N-1} = {-}\frac{\left( m_{2\cdots N}{\varvec{\nabla }}_1 - m_1{\varvec{\nabla }}_{2\cdots N}\right) ^2}{2\mu _{1|N-1}m^2_{1\cdots N}} \,. \end{aligned}$$

in position-space representation. The above expressions involve the total mass

9 $$\begin{aligned} m_{n\cdots N} = m_n+\cdots +m_{N} \end{aligned}$$

of the $n,\cdots N$ subsystem—used with $n=1$ and $n=2$ in Eq. (8)—and the reduced mass $\mu _{1|N-1}$ defined via

10 $$\begin{aligned} \frac{1}{\mu _{1|N-1}} = \frac{1}{m_1}+\frac{1}{m_{2\cdots N}} \,. \end{aligned}$$

Note that the first three terms in Eq. (7) constitute just the Hamiltonian ${\hat{H}}_{2\cdots N}$ of the ${\{2,\cdots N\}}$ subsystem with the c.m. kinetic energy removed, while the remaining part ${\hat{K}}^{\text {rel}}_{1|N-1}$ describes the relative motion of particle 1 with respect to the center of mass of the ${\{2,\cdots N\}}$ subsystem. Within *S* one can use completeness and separation of variables to expand $\psi ^B_N({\mathbf {r}}_1,\cdots {\mathbf {r}}_N)$ as a linear combination of products of eigenstates of ${\hat{H}}_{2\cdots N}$ (with total linear momentum zero) and eigenstates of ${\hat{K}}^{\text {rel}}_{1|N-1}$:

11 $$\begin{aligned} \psi ^B_N({\mathbf {r}}_1,\cdots {\mathbf {r}}_N) = \sum _\alpha \psi _\alpha ({\mathbf {r}}_2,\cdots {\mathbf {r}}_{N}) \,\chi _\alpha (\mathbf {r}_{1|N-1}) \,. \end{aligned}$$

Here ${\mathbf {r}}_{1|N-1} = {\mathbf {r}}_1 - (m_2{\mathbf {r}}_2+\cdots +m_{N}{\mathbf {r}}_{N})/{m_{2\cdots N}}$ and $\alpha$ labels states in the spectrum of ${\hat{H}}_{2\cdots N}$ (the sum in Eq. (11) is understood to include an integral if the spectrum is not entirely discrete).

The simplest scenario is given by assuming that the ground state of ${\hat{H}}_{2\cdots N}$ is a bound state with energy ${-}B_{N-1}$, wavefunction $\psi ^B_{N-1}({\mathbf {r}}_2,\cdots {\mathbf {r}}_{N})$, and vanishing total orbital angular momentum. All these assumptions will be relaxed later in the discussion. As $r_{1|N-1}=\left| \mathbf{r}_{1|N-1}\right|$ becomes large, one finds that

12 $$\begin{aligned}&\psi ^B_N({\mathbf {r}}_1,\cdots {\mathbf {r}}_N) \propto \psi ^B_{N-1}({\mathbf {r}}_2,\cdots {\mathbf {r}}_{N}) \nonumber \\&\quad \times (\kappa _{1|N-1}r_{1|N-1})^{1-d/2} \, K_{d/2-1}(\kappa _{1|N-1}r_{1|N-1}) + \cdots , \end{aligned}$$

where $K_{d/2-1}$ is a modified Bessel function of the second kind and

13 $$\begin{aligned} \kappa _{1|N-1} = \sqrt{2\mu _{1|N-1}\big (B_{N}-B_{N-1}\big )} \end{aligned}$$

is the momentum scale that characterizes this particular channel. For the excited states of the $N{-}1$ system, indicated by the ellipses in Eq. (12), there will be terms analogous to the one shown explicitly, but they are exponentially suppressed compared to the leading contribution due to the larger energy difference with $B_{N}$.

In the general case one considers the center of mass of *A* particles being separated from the remaining subsystem $N{-}A$. Without loss of generality one can choose the *A* coordinates to be ${\mathbf {r}}_1,\cdots {\mathbf {r}}_{A}$. Following steps analogous to the case $A=1$, using separation of variables in the region where the two clusters are separated by a distance larger than *R* (such that there are no inter-cluster interactions) gives the *N*-particle wavefunction as

14 $$\begin{aligned} \psi ^B_N ({\mathbf {r}}_1,\cdots {\mathbf {r}}_N) \propto \psi ^B_{A}({\mathbf {r}}_1,\cdots {\mathbf {r}}_{A}) \psi ^B_{N-A}({\mathbf {r}}_{A+1},\cdots {\mathbf {r}}_{N}) \times (\kappa _{ A|N-A} r_{A|N-A})^{1-d/2} \, K_{d/2-1}(\kappa _{A|N-A}r_{A|N-A}) \,,\nonumber \\ \end{aligned}$$

where

15a $$\begin{aligned} {\mathbf {r}}_{A|N-A}&= \frac{m_1{\mathbf {r}}_1+\cdots + m_{A}{\mathbf {r}}_{A}}{m_1+\cdots +m_A} -\frac{m_{A+1}{\mathbf {r}}_{A+1} + \cdots + m_{N}{\mathbf {r}}_{N}}{m_{A+1}+\cdots +m_N} \,, \end{aligned}$$

15b $$\begin{aligned} \frac{1}{\mu _{A|N-A}}&= \frac{1}{m_1+\cdots + m_A}+\frac{1}{m_{A+1}+\cdots +m_N} \,, \end{aligned}$$

15c $$\begin{aligned} \kappa _{A|N-A}&= \sqrt{2\mu _{A|N-A}(B_{N}-B_{A}-B_{N-A})} \,, \end{aligned}$$

and ${-}B_{A}$ and ${-}B_{N-A}$ are the ground-state energies of the *A*-particle and $(N{-}A)$-particle systems, respectively. Note that the above derivation makes the simplifying assumption that both $-B_{A}$ and $-B_{N-A}$ are the energies associated with, respectively, *A* and $N{-}A$-body bound states. If instead either of these energies is associated with a continuum threshold, then Eq. (14) remains correct only up to additional prefactors that scale as inverse powers of $\kappa _{A|N-A}r_{A|N-A}$.

Removing finally also the restriction that the relative motion between clusters have zero orbital angular momentum, the most general asymptotic wavefunction for the relative motion of the two clusters has the form

16 $$\begin{aligned} (\kappa _{ A|N-A} r_{A|N-A})^{1-d/2}\sum _{\mathbf {L}} \gamma _{\mathbf {L}} Y_{\mathbf {L}}(\hat{{\mathbf {r}}}_{A|N-A}) \times K_{\ell +d/2-1}(\kappa _{A|N-A}r_{A|N-A}) \,, \end{aligned}$$

where $Y_{\mathbf{L}}$ denotes the *d*-dimensional hyperspherical harmonics for spin representation $\ell$ (the top-level hyperspherical quantum number not otherwise indicated explicitly, see for example Ref. [51] for details) and the $\gamma _{\mathbf {L}}$ are expansion coefficients. This is exactly the same behavior as found in two-particle bound states with nonzero angular momentum, discussed for $d=2$ and $d=3$ in Refs. [8, 9]. For the one-dimensional case, $\ell =\mathbf {L}=0$ and $\ell = \mathbf {L}=1$ correspond to even and odd parity, respectively, with the $d=1$ hyperspherical harmonic being simply unity for even parity, while for odd parity it is an odd step function.

Let now $B_N(L)$ denote the binding energy of the *N*-body state of interest in a cubic periodic box of length *L*, and $B_N = B_N(\infty )$. Then the finite volume correction to the binding energy is

17 $$\begin{aligned} \varDelta B_N(L) = B_N(L) - B_N \,, \end{aligned}$$

and following steps analogous to Refs. [1, 8, 9, 37] yields that in general $\varDelta B_N(L)$ receives contributions from every possible breakup channel. However, if the *N*-particle system can be subdivided as an *A*-particle bound state and $(N{-}A)$-particle bound state in a relative $\ell = 0$ state, then from the asymptotic behavior of the wavefunction derived above it follows that the leading contribution to $\varDelta B_N(L)$ is proportional to

18 $$\begin{aligned} (\kappa _{ A|N-A}L)^{1-d/2} \, K_{d/2-1}(\kappa _{ A|N-A}L) \,. \end{aligned}$$

In addition to this there are also terms that have a larger exponential suppression, starting at $\mathcal {O}\big (\mathrm {e}^{-{\sqrt{2}\kappa L}}\big )$ for $d\ge 2$, and at $\mathcal {O}\big (\mathrm {e}^{-{2\kappa L}}\big )$ for $d=1$. These can be safely neglected except possibly at very small *L*. If the two bound states have orbital angular momentum $\ell > 0$, then the finite volume correction has the same dependence as in Eq. (18) along with subleading terms that are suppressed by powers of $\kappa _{A|N-A}L$. The functional form of these terms is exactly the same as that derived for the $N=2$ case in Refs. [8, 9], with the sign of $\varDelta B_N(L)$ oscillating with even and odd $\ell$.

For the case that either or both the *A*-particle ground state and the $(N{-}A)$-particle ground state are continuum states, the exponential dependence will be the same, except that there is an additional power law factor of $P(\kappa _{A|N-A}L)$ due to the integration over continuum states,

19 $$\begin{aligned} (\kappa _{ A|N-A}L)^{1-d/2} K_{d/2-1}(\kappa _{ A|N-A}L)P(\kappa _{A|N-A}L) \,. \end{aligned}$$

The functional form for this power law factor $P(\kappa _{A|N-A}L)$ is currently not known, except for a few analytically solvable examples considered in Ref. [49].

### Numerical Implementation

The finite-volume behavior derived above can be verified by numerical calculations. The most straightforward way to do this is to discretize the Hamiltonian (1) on a spatial lattice. To factor out the overall c.m. motion from the beginning, this can be done using directly the relative coordinates $\mathbf {x}_i$ defined in Eq. (4). Using *n* sites along each axis within a space of volume $L^d$ gives the Hamiltonian as a matrix in an $n^{d\times (N-1)}$-dimensional vector space.

For two particles (with equal mass *m* and reduced mass $\mu = m/2$) in one spatial dimension the configuration-space wavefunction can be expressed in terms of a single relative coordinate $x_1 \equiv x$. The key step in discretizing the Hamiltonian is replacing the derivative in the kinetic term by a finite-differences operator,

20 $$\begin{aligned} \frac{\partial ^2}{\partial x^2} \rightarrow D_2^{(k)} \,, \end{aligned}$$

where *k* denotes the order of the stencil. In the simplest case, $k=2$,

21 $$\begin{aligned} D_2^{(2)} \psi (x) = \frac{1}{a^2}\big [\psi (x-a) - 2\psi (x) + \psi (x+a)\big ] \,, \end{aligned}$$

where $a=L/n$ denotes the lattice spacing. Formally one can consider the wavefunction to be expanded in terms of functions exactly localized on lattice sites,

22 $$\begin{aligned} \psi (x) = \sum _{i} c_i \chi _i(x) \ \ \text {,}\ \ \chi _i(x) = \delta (x - x_i) \,, \end{aligned}$$

with $x_i = i L/n$, $i={-}n/2,\cdots n/2-1$ denoting the *i*th lattice site, assuming *n* to be even for convenience. Local potentials are trivial to handle in this framework since one merely has $V(x)\psi (x) \rightarrow V(x_i)\psi (x_i)$. Overall, this procedure gives the Hamiltonian as a very sparse matrix, with the kinetic part being tridiagonal for $k=2$ and the potential part diagonal. In general, the discretization can more elegantly be expressed in a second-quantized formalism, see for example Ref. [52] for a discussion in the context of lattice Monte Carlo methods.

The discretization is of course an approximation. Most notably, it affects the dispersion relation that relates energies and momenta. While eigenstates of the free Hamiltonian remain plane waves on the lattice,

23 $$\begin{aligned} \phi _j(x) = \langle x|\phi _j\rangle = \frac{1}{\sqrt{L}}\exp (\mathrm {i}p_j x) \,, p_j = \frac{2\pi j}{L} \,, \end{aligned}$$

applying Eq. (21) gives

24 $$\begin{aligned} {\hat{K}}\phi _j(x) \rightarrow {-}D_2^{(2)} \phi _j(x) = \frac{2}{a^2}\big [\cos (a p_j)-1]\phi _j(x) = p_j^2 \phi _j(x) + \mathcal {O}(a^2) \end{aligned}$$

for ${\hat{K}} = {\hat{K}}^{\text {rel}}_{\text {2-body}}$. The use of stencils with $k>2$, determined for any *k* as the solution of a linear equation system [53], pushes the corrections in Eq. (24) to higher orders in *a*. This increases the computational cost, however, since increasing *k* reduces the sparsity of the Hamiltonian matrix by adding bands further away from the diagonal.

The above procedure is implemented in a code that is made available as Supplemental Material along with Ref. [49]. To complement the fully generic derivation, holding for any number of particles in an arbitrary number of spatial dimensions, the implementation consists of a generator program (conveniently written in Haskell due to the highly recursive nature of the problem). This generates a script (to be run with GNU Octave or compatible software) for each desired setup. For simplicity, this program only handles the case where all particles have equal mass, but it is straightforward to adapt the Haskell code for heterogeneous systems.

**Fig. 1:**
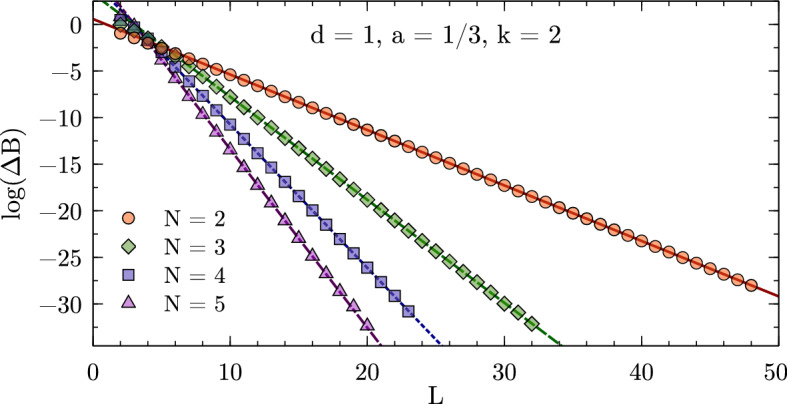
Finite-volume energy shift for $N=2,3,4,5$ particles interacting via a Gaussian potential ($R=1$, $V_0=-1$) in one dimension. All quantities are given in units of the particle mass $m=1$ (see text)

Periodic boundary conditions are implemented by using index maps for each coordinate, which also makes it very easy to generate the kinetic term in the Hamiltonian. The code supports these to *k*th order accuracy with arbitrary even $k\ge 2$.

Section 3.1 discusses a more elaborate but closely related implementation to discretize the Hamiltonian in a way that maintains the exact continuum dispersion relation. In that context it is also described how to include spin (or other discrete) degrees of freedom, as well as how to explicitly construct states with definite symmetry properties. This is useful to simulate concrete systems of physical interest, while for testing the bound-state volume dependence it suffices to run calculations using the generator code provided with Ref. [49].

### Explicit Numerical Checks

**Fig. 2:**
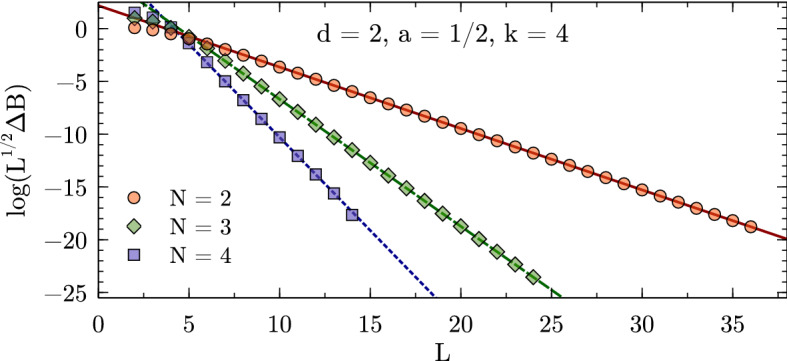
Finite-volume energy shift for $N=2,3,4$ particles interacting via a Gaussian potential ($R=1.5$, $V_0=-1.5$) in two dimensions. All quantities are given in units of the particle mass $m=1$ (see text)

**Fig. 3:**
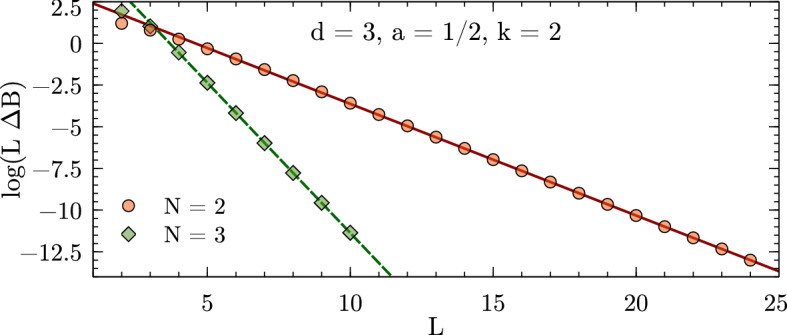
Finite-volume energy shift for $N=2,3$ particles interacting via a Gaussian potential ($R=1$, $V_0=-5$) in three dimensions. All quantities are given in units of the particle mass $m=1$ (see text)

Figures 1, 2, and 3 show numerical results for, respectively, 1, 2, and 3 spatial dimensions. These where obtained for equal-mass particles interacting via local attractive Gaussian potentials,

25 $$\begin{aligned} V(r) = V_0 \exp \biggl (-\Bigl (\frac{r}{R}\Bigr )^2\biggr ) \,. \end{aligned}$$

While these potentials do not have a strictly finite range as assumed in the derivation of the volume dependence, their fall-off at large distances is much faster than any expected volume dependence and therefore the relations remain valid up negligibly small corrections. The use of Gaussian wells instead of, e.g., strictly finite-range step potentials has the advantage of minimizing discretization artifacts, which are furthermore controlled in the kinetic part to calculations by using $k=2,4,\cdots$ finite differences for the kinetic energy. The lattice spacings *a* for the calculations are chosen to minimize discretization artifacts as much as possible while probing volumes large enough to test the asymptotic behavior of the finite-volume corrections. These calculations use natural units, which besides $\hbar =c=1$ also set the mass to unity, $m = 1$, so that all numbers are quoted without explicit dimension.

Expanding the Bessel function in Eq. (18) reveals that the leading finite-volume correction has the asymptotic exponential form

26 $$\begin{aligned} \varDelta B_N(L) \propto \exp \left( {-}\kappa _{A|N-A}L\right) / L^{(d-1)/2} \end{aligned}$$

characteristic for bound states. This form can be easily identified by plotting the logarithm of $\varDelta B_N(L)$ times $L^{(d-1)/2}$ as a function of *L*, and linear fits can be used to extract the slopes to be compared to the expected $\kappa _{A|N{-}A}$. The straight lines fitting the data points in the figures indicate excellent agreement of the numerical calculation with the expected form, and Table 1 (which also gives the particular parameters $V_0$ and *R* used for the Gaussian potential in each case) furthermore shows very good quantitative agreement for the $\kappa _{A|N{-}A}$.

**Table 1:**
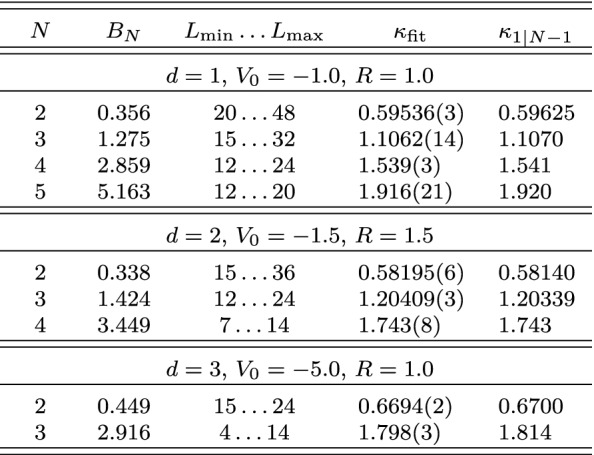
Numerical results for local Gaussian well potentials $V(r) = V_0\exp (-r^2/R^2)$

All quantities are given in units of the particle mass $m=1$ (see text)

**Fig. 4:**
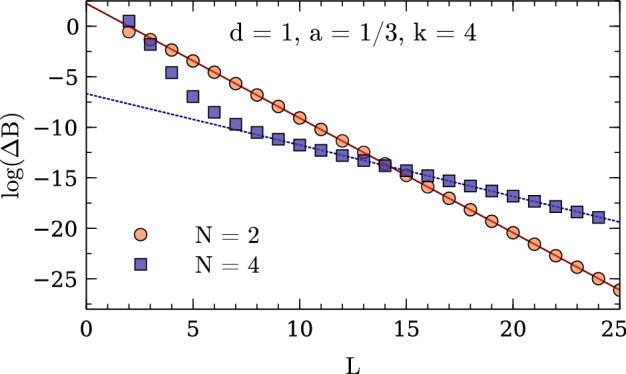
Finite-volume energy shift for $N=2$ and $N=4$ particles interacting via a Gaussian potentials in one dimension. An attractive two-body potential is supplemented by a repulsive three-body one, making the three-body system unbound, and finally by an attractive four-body potential in order to bind that system (see text for details). All quantities are given in units of the particle mass $m=1$

For all these calculations, since the interaction was chosen to be a purely attractive two-body potential, the dominant scale is $\kappa _{1|N-1}$ because all clusters of $N'<N$ particles are bound. While in general one would have to heavily fine tune a two-body interaction to create anything different from this, it is possible to introduce few-body interactions to create a situation where $N=2,4$ states are bound whereas no bound three-body state exists. For $d=1$, a concrete example is constructed by supplementing a Gaussian two-body potential with $V_0={-}2.5$ and $R=1$, generating a two-body bound state at $E={-}1.29$, with a repulsive local three-body force,

27 $$\begin{aligned} V_{3}(x_1,x_2,x_{12})= & {} V_0^{(3)} \exp \Biggl ({-}\biggl (\frac{x_1}{R_0^{(3)}}\biggr )^2\Biggr ) \nonumber \\&\exp \Biggl (-\biggl (\frac{x_2}{R_0^{(3)}}\biggr )^2\Biggr ) \exp \Biggl ({-}\biggl (\frac{x_{12}}{R_0^{(3)}}\biggr )^2\Biggr ) \,, \end{aligned}$$

where $x_{12}=|\mathbf {x}_1-\mathbf {x}_2|$, cf. Eq. (6). Setting $V_0^{(3)}=10$ and $R_0^{(3)}=2$ makes the $N=3$ system unbound, which is compensated for $N=4$ by adding an analogous short-range four-body force—using products of Gaussians in all relative pair coordinates, analogous to Eq. (27)—with $V_0^{(4)} = {-}24$ and $R_0^{(4)}$ to obtain a four-body bound state at $E={-}2.71$. The volume dependence for this system is shown in Fig. 4. While at small volumes the $N=4$ behavior is complicated (likely determined by a $2+1+1$ channel), a clear linear behavior (on the appropriate log scale) is observed asymptotically, and the extracted slope 0.508(2) is in excellent agreement with $\kappa _{2|2} = 0.502$ (considering that the quoted uncertainty is obtained from the linear fit alone).

The excellent agreement of the numerical results with the theoretical expectation, and the fact that the volume dependence is dominated by only two parameters, $\kappa _{A|N{-}A}$ and the proportionality factor not shown explicitly in Eq. (26), establishes that robust extrapolations to infinite volume can be obtained from a small set of small volumes. Beyond that fact, knowing the functional form of the volume dependence is useful in a more direct way because the proportionality factor is directly related to the asymptotic normalization coefficient (ANC) associated with the $A+(N{-}A)$ threshold. ANCs play an important role for low-energy capture processes that govern nucleosynthesis in stellar environments [54–57] and are notoriously difficult to extract experimentally due to the dominance of the Coulomb repulsion at low energies.

In the limit where the separation distance $r_{A|N{-}A}$ between the two clusters is large, the normalized *N*-body wavefunction is a product of normalized *A*-body and $(N{-}A)$-body wavefunctions times the relative wavefunction as written in Eq. (16). The ANC is then the coefficient $\gamma _\mathbf{L}$ in Eq. (16), which is shortened to just $\gamma$ in the following. For the case of $d=2$, where for a precise determination of the ANC it is most convenient to *not* expand the Bessel functions as in Eq. (26) because that expansion discards some logarithmic corrections in $d=2$ dimensions. Note that this leads to a definitions which slightly differs from the one used in Ref. [9]. Numerically, the relative wavefunction can be obtained by calculating the ratio

28 $$\begin{aligned} \left( \frac{\langle \varPsi ^B_N| O_A(\mathbf {r}_{A|N-A}) O_{N-A}(\mathbf {0}) |\varPsi ^B_N\rangle }{\langle \varPsi ^B_A| O_A(\mathbf {0}) |\varPsi ^B_{A}\rangle \langle \varPsi ^B_{N-A}| O_{N-A}(\mathbf {0}) |\varPsi ^B_{N-A}\rangle }\right) ^{\!1/2} \end{aligned}$$

for some localized *A*-body and $(N{-}A)$-body operators $O_{A}(\mathbf {r})$, $O_{N-A}(\mathbf {0})$. The result of this determination can then be compared to the the asymptotic form as given in Eq. (16), taking into account additional copies due to the periodic boundary conditions. The ratio gives the magnitude of the ANC, denoted by $\left| \gamma \right| _{\text {WF}}$ to indicate the determination directly from the wavefunction.

In addition, the ANC can be obtained in a completely different way using the finite-volume correction $\varDelta B_N(L)$. By combining the *N*-body results summarized here with the derivations in Refs. [1, 8, 9], one finds that $\varDelta B_N(L)$ equals

29 $$\begin{aligned} \frac{(-1)^{\ell +1} \sqrt{\tfrac{2}{\pi }} f(d)\left| \gamma \right| ^2}{\mu _{A|N-A}} \kappa ^{2-d/2}_{A|N-A}L^{1-d/2} K_{d/2-1}(\kappa _{A|N-A}L) \,, \end{aligned}$$

plus corrections that are exponentially suppressed further. This relation follows directly from defining the ANC in terms of the asymptotic radial wavefunction, which for a cluster separation $r_{A|N-A}$ large compared to the range of the interaction is universally given by

30 $$\begin{aligned} \psi _{\text {asympt}}(r_{A|N-A}) = \gamma \, \sqrt{\frac{2\kappa _{A|N-A}}{\pi }} (r_{A|N-A})^{1-d/2} K_{d/2-1}(\kappa _{A|N-A}r_{A|N-A}) \, Y(d) \,, \end{aligned}$$

where *Y*(*d*) accounts for the angular normalization in *d* spatial dimensions. For $d=3$, where $Y(3) = 1/\sqrt{4\pi }$, the convention in Eq. (30) reproduces the canonical form

31 $$\begin{aligned} \gamma \exp ({-}\kappa _{A|N-A}r_{A|N-A})/r_{A|N-A} \end{aligned}$$

for a two-cluster S-wave state. For $d=1$ one has $Y(1) = 1$ and the asymptotic form is simply $\gamma \exp ({-}\kappa _{A|N-A}r_{A|N-A})$, while as already stated for $d=2$ it is more natural to define the ANC directly in terms of the modified Bessel function, which does not fully reduce to a simple exponential in this case. The function *f*(*d*) captures these conventional differences and takes values $f(1)=2$, $f(2)=\sqrt{8/\pi }$, and $f(3)=3$. For $d=3$, $\varDelta B_N(L)$ is averaged over all $2\ell +1$ elements of a given angular momentum $\ell$ multiplet, while for $d=2$ the average is taken over symmetric and antisymmetric combinations of $\mathbf {L} = \pm \ell$ for even $\ell$ [9]. The result of this ANC extraction, using fits of Eq. (29) to the data shown in Figs. 1, 2, 3, is denoted as $\left| \gamma \right| _{\text {FV}}$. Note that if there are several different ways to partition the *N*-particle system into clusters with equal $\kappa _{A|NA}$, then there will contributions to the finite-volume correction from each channel.

Using the same Gaussian well potentials as discussed previously, results for $\left| \gamma \right| _{\text {FV}}$ and $\left| \gamma \right| _{\text {WF}}$ are shown in Table 2. This analysis used Eq. (28) with the operator $O_1$ equal to the single particle density and $O_{N-1}$ equal to the $(N-1)$-body density on a single lattice site, with all quantities extracted at the same finite volume. As seen in Table 2, the two methods for extracting the ANCs are in excellent agreement. The technique therefore provides a strikingly simple and robust way to extract ANCs, which will be of great practical relevance once an extension of the finite-volume formalism to include the Coulomb force is available. Finally, it is worth noting that with $\left| \gamma \right| _{\text {WF}}$ extracted from a single volume (assuming one is using a method that gives access to the wavefunction), one can in fact determine $B_N(L)$ from a single-volume calculation [49]. This can be relevant in practice for cases where calculations at multiple volumes are prohibitively expensive.

**Table 2:**
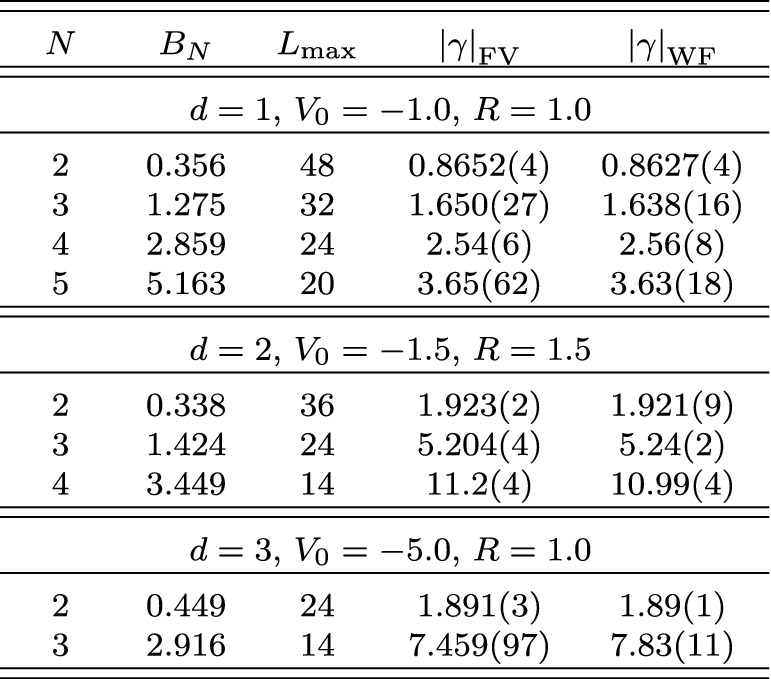
Extracted ANCs for local Gaussian well potentials $V(r) = V_0\exp (-r^2/R^2)$

All quantities are given in units of the particle mass $m=1$

## Resonances

All levels in the discrete finite-volume energy spectrum that are not bound states—characterized by the asymptotic exponential behavior discussed in Sect. 2—have a power-law volume dependence. The Lüscher formalism used to extract infinite-volume scattering observables is based on analyzing how these levels are shifted by the interaction among the particles compared to the free (non-interacting) energy levels [2, 3]. As mentioned in the introduction, resonance states do not correspond to individual energy levels at finite volume, but instead are manifest as (sequences of) avoided crossings within the power-law spectrum. This is well established for two-body systems [4–6], whereas Ref. [50] showed that this result carries over to the few-body sector, thereby establishing finite-volume calculations as a theoretical tool to discover resonances that can be interpreted as metastable states of $N\ge 3$ constituents. These results, and in particular the “discrete variable representation (DVR)” used as numerical method for these calculations, are discussed in the following.

### Discrete Variable Representation

The starting point for the DVR construction used here are plane-wave states $\phi _j(x)$, where $j={-}n/2,\cdots n/2-1$ for $n>2$ even, defined in Eq. (23), where *x* at this point denotes a single relative coordinate in one dimension. It is clear that any periodic solution of the Schrödinger equation can be expanded in terms of these states, and this expansion becomes exact for $n\rightarrow \infty$.

Following the general construction described in Ref. [58], consider now pairs $(x_k, w_k)$ of quadrature points $x_k$ and associated weights $w_k$ such that

32 $$\begin{aligned} \sum \limits _{k={-}n/2}^{n/2-1} w_k\,\phi _i^*(x_k) \phi _j(x_k) = \delta _{ij} \,. \end{aligned}$$

For the plane-wave states (23), this is satisfied by an equidistant mesh with constant weight:

33 $$\begin{aligned} x_k = \frac{L}{n}k \ \ \text {,}\ \ w_k = \frac{L}{n} \,\forall k \,. \end{aligned}$$

**Fig. 5:**
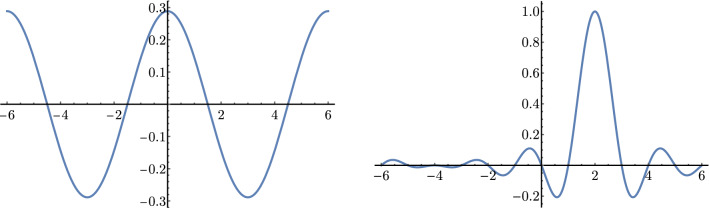
Plane-wave (left) and DVR states (right) for a single relative coordinate *x* in one dimension. What is plotted are the real parts of the wavefunctions

Equipped with this one can define matrices

34 $$\begin{aligned} {\mathcal {U}}_{ki} = \sqrt{w_k} \phi _i(x_k) \,, \end{aligned}$$

and these matrices are unitary by Eq. (32). The DVR basis functions $\psi _k(x)$ are defined by rotating the original plane-wave basis with ${\mathcal {U}}^*$, where the asterisk denotes complex conjugation:

35 $$\begin{aligned} \psi _k(x) = \sum \limits _{i={-}n/2}^{n/2-1} {\mathcal {U}}^*_{ki} \phi _i(x) \end{aligned}$$

for $k = {-}n/2,\cdots n/2-1$. The range of indices is the same as for the original plane-wave states, but whereas in Eq. (23) they specify a momentum mode, $\psi _k(x)$ is approximately localized at position $x_k \in [{-}L/2,L/2\big )$. An example is shown in Fig. 5.

An explicit evaluation of Eq. (35) reveals that

36 $$\begin{aligned} \psi _k(x) = \sum \limits _{i={-}n/2}^{n/2-1} \phi _i(x-x_k) = \frac{1}{\sqrt{L}} \sum \limits _{i={-}n/2}^{n/2-1} \mathrm {e}^{\mathrm {i}p_i(x-x_k)} \,, \end{aligned}$$

so the DVR construction can be related to a discrete Fourier transform (DFT).^1^ Indeed, as seen in the right panel of Fig. 5, $\psi _k(x)$ approximates a delta function centered at $x_k$, and from the definitions one finds that

37 $$\begin{aligned} \psi _k(x_j) = \frac{1}{\sqrt{w_k}} \delta _{kj} \,. \end{aligned}$$

This establishes a close relation to the discretization discussed in Sect. 2.1. Within the space of DVR states, the dispersion relation is exact (formally one can think of achieving this with an infinite-order finite-difference method for the derivative), and in line with this the kinetic energy is given by a dense matrix:

38 $$\begin{aligned} \langle \psi _k|{\hat{K}}|\psi _l\rangle = {\left\{ \begin{array}{ll} \dfrac{\pi ^2 N^2}{6\mu L^2} \left( 1+\dfrac{2}{n^2}\right) \,, &{}\text {for}\quad k = l\,, \\ \dfrac{({-}1)^{k-l} \pi ^2}{\mu L^2 \sin ^2\big (\pi (k-l)/n\big )}\,, &{}\text {otherwise}\,. \end{array}\right. } \end{aligned}$$

This is more computationally demanding than the band-diagonal structure obtained from a simple finite-difference discretization, but still the matrix elements are known explicitly for any index pair (*k*, *l*). More importantly, for $d>1$ the matrix becomes sparse, as will be seen below. Alternatively, as pointed out in Ref. [61], one can exploit the relation of the plane-wave based DVR to the DFT and evaluate the kinetic energy in momentum space. This involves transforming to the original plane-wave basis (23), applying

39 $$\begin{aligned} {\hat{K}}|\phi _i\rangle = \frac{p_i^2}{2\mu }|\phi _i\rangle \,, \end{aligned}$$

and then transforming back.

Importantly, evaluation of (local) potential matrix elements is just as simple as with the discretization of Sect. 2.1. From Eq. (37) it follows that

40 $$\begin{aligned} \langle \psi _k|{\hat{V}}|\psi _l\rangle&= \int \mathrm {d}x\,\psi _k^*(x) V(x) \psi _l(x)\, \nonumber \\&\approx \sum \limits _{i={-}n/2}^{n/2-1} w_m\,\psi _k^*(x_i) V(x_i) \psi _l(x_i)\ = V(x_k) \delta _{kl} \,, \end{aligned}$$

so that the potential operator is diagonal in the DVR representation. The approximation indicated in the second line in Eq. (40) lies in replacing the integral by a sum, which is possible because the $(x_k,w_k)$ defined in Eq. (33) constitute the mesh points and weights of a trapezoidal quadrature rule.^2^ While not very accurate in general, this quadrature rule is highly efficient for integrating periodic functions.

#### General Construction

The construction is straightforward to generalize to the case of an arbitrary number of particles *N* and spatial dimensions *d*, starting from product states of $(N-1)\times d$ plane waves, one for each relative-coordinate component. The transformation matrices and DVR basis functions are defined via tensor products, and DVR states are labeled by a collection of $(N-1)\times d$ indices. Using the short-hand notation $|\psi _k\rangle = |k\rangle$, a general state is written as

41 $$\begin{aligned} |s\rangle = | (k_{{1,1}},\cdots k_{{1,d}}),\cdots ,(k_{{N-1,1}},\cdots k_{{N-1,d}}); (\sigma _1,\cdots ,\sigma _N) \rangle \,, \end{aligned}$$

including additional indices $\sigma _i$ to account for spin degrees of freedom. If the particles have spin *S*, then each $\sigma _i$, labeling the projections, takes values from ${-}S$ to *S*. Additional internal degrees of freedom, such as isospin, can be included in the same way. The space spanned by all these states $|s\rangle$ is denoted by *B*.

As already mentioned, the kinetic energy becomes a sparse matrix for $d>1$. A one-dimensional matrix element (38) enters for each component $k_{i,c}$, multiplied by Kronecker deltas for all $c' \ne c$ and summed for all relative coordinates $i=1,\ldots ,N-1$. Working with simple relative coordinates as defined in Eq. (4) implies that the general kinetic energy operator,

42 $$\begin{aligned} {\hat{K}}^{\text {rel}}_{\text {{ N}-body}} = {-}\frac{1}{2\mu } \sum \limits _{i=1}^{N-1}\sum \limits _{j=1}^i \frac{\partial }{\partial x_i}\frac{\partial }{\partial x_j} \,, \end{aligned}$$

contains mixed (non-diagonal) terms, e.g.,

43 $$\begin{aligned} {\hat{K}}^{\text {rel}}_{\text {3-body}} = -\frac{1}{2\mu }\biggl (\frac{\partial ^{2}}{\partial x_1^{2}}+\frac{\partial ^{2}}{\partial x_2^{2}}+\frac{\partial }{\partial x_1}\frac{\partial }{\partial x_2}\biggr ) \end{aligned}$$

for three particles in one dimension. The kinetic-energy matrix elements are in this case given by

44 $$\begin{aligned} \langle k_{1}k_{2}|{\hat{K}}^{\text {rel}}_{\text {3-body}}|l_{1}l_{2}\rangle = \langle k_1|{\hat{K}}^{\text {rel}}_{x_1}|l_1\rangle \delta _{k_2 l_2} + \langle k_2|{\hat{K}}^{\text {rel}}_{x_2}|l_2\rangle \delta _{k_1 l_1} + \langle k_{1}k_{2}|{\hat{K}}^{\text {rel}}_{x_1-x_2}|l_{1}l_{2}\rangle \,, \end{aligned}$$

where the first two matrix elements on the right-hand side are given in Eq. (38) and the last term is a special case of the general mixed-derivative matrix element

45 $$\begin{aligned} \langle k_{i}k_{j}|{\hat{K}}^{\text {rel}}_{x_i-x_j}|l_{i}l_{j}\rangle = {-}\frac{1}{2\mu } \big [\langle k_i|\partial _i|l_i\rangle \langle k_j|\partial _j|l_j\rangle \big ] \end{aligned}$$

with [62]

46 $$\begin{aligned} \langle k|\partial |l\rangle = {\left\{ \begin{array}{ll} {-}\mathrm {i}\dfrac{\pi }{L}\,, &{}\text {for}\quad k = l\,, \\ \dfrac{\pi }{L} \dfrac{({-}1)^{k-l}\exp \!\left( {-}\mathrm {i}\dfrac{\pi (k-l)}{n}\right) }{\sin \!\left( \dfrac{\pi (k-l)}{n}\right) }\,, &{}\text {otherwise}\,. \end{array}\right. } \end{aligned}$$

As for the diagonal terms, for a general state $|s\rangle$ such terms are summed over for all pairs of relative coordinates and spatial components *c*, including Kronecker deltas for $c' \ne c$.

#### Reduction by Symmetry

In the form introduced above, the DVR basis includes states with many different symmetry properties. To focus on a particular sector of interest, there are different possibilities to single out subspaces of the full Hilbert space that the truncated basis approximates. The most direct approach explicitly constructs linear combinations of states with the desired properties. It is a feature of the DVR basis that for some important cases this construction can be carried out with great efficiency.

To study systems of identical bosons (or fermions) it is necessary to consider subspaces of states which are fully (anti-)symmetric under permutations of the individual particles. A convenient way to construct such states follows the method described in Ref. [63]. While that paper considers the stochastic variational model in Jacobi coordinates, it is straightforward to adapt the procedure for DVR states expressed in simple relative coordinates. The relevant steps are as follows:

1. The transformation from single-particle to relative coordinates (and *vice versa*) is constructed as given in Eq. (5)
2. For the *N*-particle system there are *N*! permutations, constituting the symmetric group $S_N$. A permutation $p \in S_N$ can be represented as a matrix *C*(*p*) with
  47 $$\begin{aligned} C(p)_{ij} = {\left\{ \begin{array}{ll} 1\,, &{}\text {for}\quad j = p(i)\,, \\ 0\,, &{}\text {otherwise}\,, \end{array}\right. } \end{aligned}$$
  acting on the single-particle coordinates $\mathbf {r}_i$.
3. The operation of $p \in S_N$ on the relative coordinates is then given by the matrix
  48 $$\begin{aligned} C_{\text {rel}}(p) = U\,C(p)\,U^{{-1}} \,, \end{aligned}$$
  with the last row and column of the left-hand side, corresponding to the overall c.m. coordinate, discarded, so that $C_{\text {rel}}(p)$ is an $(N{-}1)\times (N{-}1)$ matrix.

Since the indices $k_{i,c}$ correspond directly to positions on the spatial grid used to define the initial plane-wave states (recall that for a two-body system in one dimension $\psi _k(x)$ is peaked at $x=x_k$), acting with $C_{\text {rel}}(p)$ on a state $|s\rangle$ is now straightforward: the $k_{i,c}$ are transformed according to the entries $C_{\text {rel}}(p)_{ij}$, where for each *i* one considers all $c=1,\ldots ,d$ at once. In other words, $C_{\text {rel}}(p)$ is expanded (by replication for each *c*) to a matrix acting in the space of individual coordinate components. As a final step, to maintain periodic boundary conditions, any transformed indices that may fall outside the original range ${-}n/2,\ldots ,n/2-1$ are wrapped back into this interval by adding appropriate multiples of *n*. Applying the permutation to the spin indices $(\sigma _1,\ldots ,\sigma _N)$ is trivial because they are given directly as an *N*-tuple. The final result of this process for a given state $|s\rangle \in B$ and permutation *p* is a transformed state,

49 $$\begin{aligned} |s'\rangle = {\mathcal {C}}(p) |s\rangle \in B \,, \end{aligned}$$

where

50 $$\begin{aligned} {\mathcal {C}}(p) = C_{\text {rel}}(p) \, C_{\text {spin}}(p) \end{aligned}$$

denotes the total permutation operator in the space of DVR states. The statement of Eq. (49) is that each $p\in S_n$ acts on *B* as a whole by permuting the order of elements.

With this, one finds the symmetrization and antisymmetrization operators as

51 $$\begin{aligned} {\mathcal {S}} = \frac{1}{n!}\sum _{p\in S_n} {\mathcal {C}}(p) \ \ \text {and}\ \ {\mathcal {A}} = \frac{1}{n!}\sum _{p\in S_n} \mathrm {sgn}(p)\,{\mathcal {C}}(p) \,, \end{aligned}$$

where $\mathrm {sgn}(p) = \pm 1$ denotes the parity of the permutation *p*. Since both of these operators are projections (${\mathcal {S}}^2 = {\mathcal {S}}$, ${\mathcal {A}}^2 = {\mathcal {A}}$), they map the original basis *B* onto bases $B_\mathcal {S/A}$ of, respectively, symmetrized or antisymmetrized states, each of which consists of linear combinations of states in *B*.

An important feature of these mappings is that each $|s\rangle \in B$ appears in at most one state in $B_{\mathcal {S}}$ (for symmetrization) or $B_{\mathcal {A}}$ (for antisymmetrization). Thus, to determine $B_\mathcal {S/A}$ it suffices to apply $\mathcal {S/A}$ to all $|s\rangle \in B$, dropping duplicates which occur when the operator is applied to a state in *B* that has already been generated by a previous permutation. This algorithm is straightforward to apply in numerical calculations and it can be made highly efficient. Moreover, computer memory can be saved by storing for each combined state in $B_\mathcal {S/A}$ only the index of one state in *B* that generates it. This can be useful to trade memory efficiency against an increase in computational time since the coefficients defining the combined states have to be recalculated as needed.

*Cubic symmetry* While permutation symmetry and parity remain unaffected by the finite volume, rotational symmetry (for $d>2$) is explicitly broken by the periodic box. In $d = 3$ dimensions (to which the remaining discussion in this section will be limited), angular momentum $\ell$ associated with spherical *SO*(3) symmetry is no longer a good quantum number. Instead, one has to consider the breaking of *SO*(3) down to a cubic subgroup $\mathcal {O}\subset SO(3)$.

This group has 24 elements and five irreducible representations $\varGamma$, conventionally labeled $A_1$, $A_2$, *E*, $T_1$, and $T_2$. Their dimensionalities are 1, 1, 2, 3, and 3, respectively, and irreducible representations $D^l$ of *SO*(3), determining angular-momentum multiplets in the infinite volume, are reducible with respect to $\mathcal {O}$. As a consequence, any given angular-momentum state in infinite volume can contribute to several representations $\varGamma$. In the cubic finite volume one finds the spectrum decomposed into multiplets with definite $\varGamma$, where an index $\alpha =1,\ldots ,\dim \varGamma$ further labels the states within a given multiplet.

For the calculations considered here, it is desirable to select spectra by their cubic transformation properties. To that end, one constructs projection operators [64],

52 $$\begin{aligned} {\mathcal {P}}_\varGamma = \frac{\dim \varGamma }{24} \sum _{R\in \mathcal {O}}\,\chi _\varGamma (R) D_{n}(R) \,, \end{aligned}$$

where $\chi _\varGamma (R)$ denotes the character (tabulated in Ref. [64]) of the cubic rotation *R* for the irreducible representation $\varGamma$ and $D_{n}(R)$ is the realization of the cubic rotation in our DVR space of periodic *n*-body states. For example, for the one-dimensional representation $\varGamma =A_1$, $\chi _{A_1}(R)=1$ for all cubic rotations *R*, so in this case Eq. (52) reduces to an average over all rotated states, analogous to how one can project onto *S* waves ($\ell =0$) in infinite volume. The construction of the $D_{n}(R)$ is discussed in detail in Ref. [50].

### Numerical Implementation

The DVR scheme described above essentially amounts to constructing the Hamiltonian ${\hat{H}}$ for the physical system of interest in a particular truncated basis (*B*), which then gives rise to a finite matrix representation $H={\hat{H}}\big |_B$. The dimension of this matrix grows with (i) the number *N* of particles (and their spin as well as potential other discrete degrees of freedom), (ii) the number *d* of spatial dimensions, and (iii) the number *n* of DVR states used in the calculation. The precise scaling for particles with spin *S* is

53 $$\begin{aligned} \dim B = (2S+1)^N \times n^{(N-1)\times d} \,. \end{aligned}$$

*N*, *S*, and *d* are fixed by the particular physics problem, but the appropriate choice of *n* depends on all other parameters as well as on details of the interaction and the size *L* of the volume. While in principle *n* has to be sufficiently large to converge both the kinetic and the potential parts entering the Hamiltonian, the most important effect comes from Eq. (40): the representation of the (local) interaction as a diagonal matrix rests on the quality of the quadrature that approximates the integral. The more peaked (or, generally, more “structured”) the shape of the potential, the larger *n* needs to be to adequately approximate the integral, and likewise, the larger *L*, the larger *n* is needed since the spacing between quadrature points increases with *L* at fixed *n*. In practice, a sequence of increasing *n* has to be considered at each volume until sufficient convergence is reached, i.e., until the change of energies with *n* is negligible compared to the desired precision. It is worth noting that all symmetries discussed in Sect. 3.1.2 are by construction exact at each *n*, so there are no convergence issues as far as that part of the calculation is concerned. Finally, the whole setup discussed in this section could be adapted to use the simple finite-difference discretization described in Sect. 2.1. This would render the kinetic-energy matrix significantly more sparse at the expense of sacrificing the exact continuum dispersion relation. In order to reach very large volumes, this may be a good tradeoff.

### Results for Few-Body Resonances

In the two-particle sector it is well established [4–6] that resonance states are manifest as avoided level crossings in the volume dependent energy spectrum $E_i(L)$, where *i* is an index labeling the discrete states in the box. Ref. [50] found that shifted Gaussian potentials,

54 $$\begin{aligned} V(r)=V_0 \exp \biggl (-\Bigl (\frac{r-a}{R_0}\Bigr )^2\biggr ) \,, \end{aligned}$$

are well suited to generate narrow resonances without much need to fine tune the parameters of the potential and furthermore showed that fitting the inflection points of individual level curves which form a plateau near the avoided crossing gives excellent agreement for the resonance energies $E_R$ with determinations from scattering phase shifts or from direct determinations as S-matrix poles on the unphysical energy sheet.

This method also works well for three-particle systems, as established in Ref. [50] by comparing to a known case from the literature. For three identical spin-0 bosons with mass $m=939.0~\mathrm {MeV}$ (mimicking neutrons) interacting via the two-body potential,

55 $$\begin{aligned} V(r) = V_0 \exp \biggl (-\Bigl (\frac{r}{R_0}\Bigr )^2\biggr ) + V_1 \exp \biggl (-\Bigl (\frac{r-a}{R_1}\Bigr )^2\biggr ) \,, \end{aligned}$$

where $V_0=-55~\mathrm {MeV}$, $V_1=1.5~\mathrm {MeV}$, $R_0=\sqrt{5}~\mathrm {fm}$, $R_1=10~\mathrm {fm}$, and $a=5~\mathrm {fm}$, it is known that a resonance state exists at $E_R=-5.31~\mathrm {MeV}$, with a half width of $0.12~\mathrm {MeV}$ [65], in addition to a two-body bound state at $E=-6.76~\mathrm {MeV}$ [66] and a three-boson bound state at $E=-37.35~\mathrm {MeV}$ [65]. Ref. [66] obtained $E=-37.22~\mathrm {MeV}$ for this bound state and $E_R=-5.96~\mathrm {MeV}$ and $\varGamma /2=0.40~\mathrm {MeV}$ for the three-body resonance, which can be understood by noting that in this calculation the potential (55) was truncated to relative *S* waves between pairs [67].

**Fig. 6:**
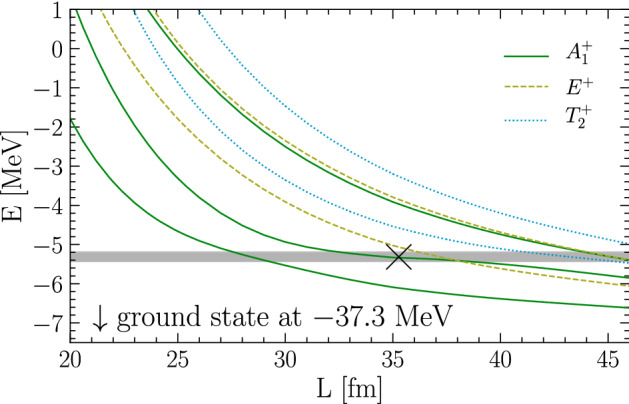
Energy spectrum of three bosons in finite volume for different box sizes *L* interacting via the potential given in Eq. (55). States corresponding to the irreducible representation $A_1$ of the cubic symmetry group are shown as solid lines, whereas $E^+$ and $T_2^+$ states are indicated as dashed and dotted lines, respectively. The shaded area indicates the resonance position and width as calculated in Ref. [65], whereas the cross marks the inflection point used here to extract the resonance energy (see text)

Using Eq. (55) in a DVR calculation gives $E=-6.756(1)$ and $E=-37.30(5)$ for the two- and three-boson ground states, respectively, in good agreement with the results of Refs. [65, 66]. These states both exhibit the exponential volume dependence as discussed in Sect. 2. In order to look for the three-boson resonance, the positive-parity three-body spectrum is calculated as a function of *L*, shown in Fig. 6. These calculations used $n=26$ DVR points at smaller volumes, and up to $n=30$ for box sizes $L\sim 40~\mathrm {fm}$ to obtain sufficiently converged results. Figure 6 furthermore indicates the cubic-group irreducible representations that individual levels belong to. These assignments were determined by running a set of cubic-projected calculations at selected volumes, while for computational efficiency the bulk of the calculation did not use the cubic projection. In general, it suffices to check the symmetry before and after each crossing observed in the spectrum to determine whether or not it is an avoided crossing (which only occurs between levels with the same quantum numbers) or an actual one.

The levels corresponding to $A_1^+$ states show an avoided crossing at about the expected resonance energy from Ref. [65], which is indicated in Fig. 6 as a shaded horizontal band, the width of which corresponds to $E_R\pm \varGamma /2$. For the other states (with quantum numbers $E^+$ and $T_2^+$) shown in the figure, no avoided crossings or plateaus are observed in the plotted range of volumes. At $L\sim 38~\mathrm {fm}$ there is an actual crossing between $A_1^+$ and an $E^+$ levels. The resonance energy is extracted from the avoided crossing by the inflection-point method, fitting polynomials

56 $$\begin{aligned} E(L) = \sum _{k=0}^{k_\text {max}} c_k L^k \,, \end{aligned}$$

to the $A_1^+$ curves participating in the avoided crossing. This procedure gives $E_R = -5.32(1)~\mathrm {MeV}$, with the uncertainty stemming from the fact that the lower level, which does not exhibit a very pronounced plateau shape, does not constrain the fit very well. The combined result from both energy levels however gives excellent agreement with the resonance energy determined in Ref. [65]. This is a clear indication that the method gives robust access to few-body resonance energies.

**Fig. 7:**
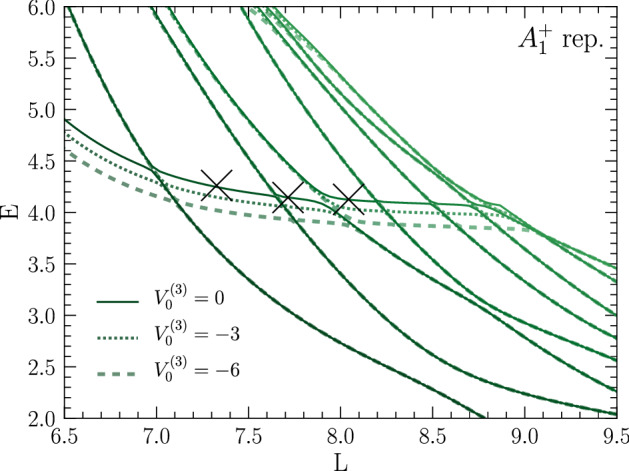
Energy spectrum of three bosons in finite volume for different box sizes *L*. The solid line shows the spectrum for three bosons interacting purely via the shifted Gaussian potential given in Eq. (54) with $V_0=2.0$ while the dashed and dotted lines show results with an additional attractive three-body force as in Eq. (27). With increasing three-body force the avoided level crossing is shifted to lower energy, while the rest of the spectrum remains unaffected

Finding good agreement with Ref. [65] establishes the validity and quantitative accuracy of the finite-volume method to extract three-body resonances. However, given that that the potential (55) supports a two-body bound state, and moreover the closeness of the resonance energy to that threshold, the three-body resonance it generates should be considered an effective two-body phenomenon. To assess the method for the discovery of “genuine” three-body resonance, i.e., states with no two-body decay channel, it is useful to consider a shifted Gaussian potential as given in Eq. (54), which does not support any two-body bound states. A three-body spectrum for this case, using $V_0=2.0$, $a=3$, and $R_0=1.5$, is shown in Fig. 7. This spectrum, completely projected onto $A_1^+$ quantum numbers, features a pronounced sequence of avoided level crossings between $E=4.0$ and $E=4.5$. Using the same inflection-point fit method as discussed above, one extracts $E_R = 4.18(8)$ as a potential resonance energy by using the three points marked with crosses in Fig. 7. In addition to this, there are several avoided crossings at lower energies that have a significant slope with respect to changes in the box size. These are interpreted as two-body resonances—known to exist at $E_{R} \sim 1.6$ for this potential [50]—embedded into the three-body spectrum. This hypothesis can be validated by repeating the calculation with an added short-range three-body force, as given in Eq. (27), setting $R_0^{(3)}=1.0$ and varying $V_0^{(3)}$. Using a set of negative values for $V_0^{(3)}$ (indicated in Fig. 7) leaves the lower avoided crossings—and in fact most of the *L*-dependent spectrum—unaffected, whereas the upper plateau set is moved downwards as $V_0^{(3)}$ is made more negative. Since the range $R_0^{(3)}=1.0$ was chosen small compared to the box sizes considered, one should indeed expect it to primarily affect states that are localized in the sense that their wavefunctions are confined to a relatively small region in the finite volume. Interpreting a resonance as a nearly bound state, its wavefunction should satisfy this criterion, whereas three-body scattering states or states where only two particles are bound or resonant are expected to have a large spatial extent. This intuitive picture gives confidence that indeed a genuine three-body resonance is seen in Fig. 7.

**Fig. 8:**
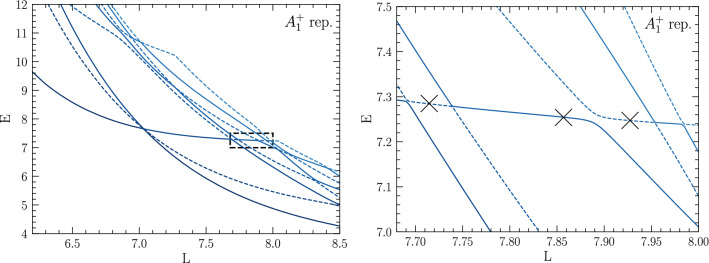
Energy spectrum of four bosons in finite volume for different box sizes *L* interacting via the shifted Gaussian potential given in Eq. (54) with $V_0=2.0$. The dashed rectangle in the left panel indicates the zoomed region shown in the right panel. All crossings are avoided because the spectrum is fully projected on states with the same quantum numbers. The crosses mark the inflection points used to extract the resonance energy (see text)

**Fig. 9:**
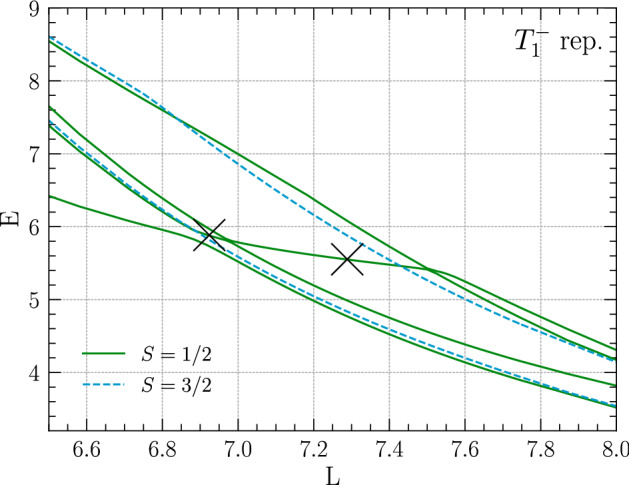
Negative-parity energy spectrum of three fermions in finite volume for different box sizes *L* interacting via the shifted Gaussian potential given in Eq. (54) with $V_0=2.0$. All levels shown in the plot were found to belong to the $T_1^-$ cubic representation by performing fully projected calculations at selected volumes. Results are shown in the spin $S=1/2$ and $S=3/2$ channels. The crosses mark the inflection points used to extract the resonance energy (see text)

Similar features are found for calculations with the same shifted Gaussian potential of four-boson and three-fermion systems. These are shown, respectively, in Figs. 8 and 9, with resonance energies extracted as $E_R^{\text {4b}}=7.26(2)$ and $E_R^{\text {3f}}=5.7(2)$. For the three-fermion calculation one needs to take into account that the overall antisymmetry of the wavefunction can be realized via different combinations of spin and spatial parts. For negative parity one finds the six lowest levels, shown in Fig. 9, to all belong to the $T_1^-$ cubic representation, which in this case has been determined by running calculations with full cubic projections at selected volumes while otherwise only restricting the overall parity. Since the interaction is spin independent, total angular momentum $\ell$ and spin *S* are separately good quantum numbers in infinite volume, and in the finite volume one likewise has $\varGamma$ and *S* to characterize states. The latter, which can be $S=1/2$ or $S=3/2$ for three spin-1/2 fermions, is determined by running calculations with fixed spin *z*-component at selected volumes, which can be realized by restricting the set of DVR basis states. Since $S=3/2$ states show up with both $S_z=3/2$ and $S_z=1/2$, whereas $S=1/2$ states are absent for $S_z=3/2$, one finds that four of the six levels shown in Fig. 9 have $S=1/2$, whereas the other two (given by the dashed lines in Fig. 9) have $S=3/2$. The resonance signature is found for $S=1/2$ in this case.

## Summary and Outlook

Finite-volume calculations provide an intriguing way to study physical systems. Their infinite-volume properties are encoded in the response of discrete energy levels to variations in the size of the volume: it is the physical S-matrix what governs the precise form of the volume dependence and therefore by studying the latter one can infer properties of the former.

For bound states this relation is manifest as asymptotic wavefunctions, characterized by their exponential fall-off scale and asymptotic normalization constants (which by analyticity are related to the S-matrix), determining the volume dependence in a direct manner. Knowing the precise form of this dependence enables controlled extrapolations of *N*-body states from small to infinite volume. This is relevant in nuclear physics for Lattice QCD [68–76] as well as Lattice EFT [21–23] calculations, and more broadly for finite-volume calculations of for example bound cold atomic systems. Furthermore, the finite-volume relations provide a direct way to calculate asymptotic normalization coefficients, which play an important role for low-energy capture processes in nuclear astrophysics and are notoriously difficult to determine experimentally. Since most of these reactions involve charged particles, future work which extends the relations to include the long-range Coulomb interaction will open the door to many interesting applications. Beyond this, studying bound states for which the nearest breakup threshold involves a splitting into more than two clusters requires more research in order to understand the additional power-law factors that arise from continuum effects and are so far known only for a few specific cases [37, 49].

Resonances are found to be robustly manifest as avoided level crossing in the spectrum as the size of the box is varied. The work discussed here establishes that this methods is able, at a quantitative level, to extract few-body resonance energies and therefore provides a discovery tool for states which are otherwise very difficult to tackle. This is important in light of much disagreement in the literature regarding the possibility of three- and four-neutron resonances from a theoretical perspective [25–33]. While such determinations are made difficult by the fact that conjectured resonance states have supposedly very small energies—requiring converged DVR calculations in large boxes due to the power-law behavior the finite-volume energy levels—carrying out such calculations can provide valuable insights regarding the existence of such exotic nuclear states. Apart from that, the finite-volume technique provides an interesting and conceptually straightforward way to study other resonances as well, such as for example the Hoyle state in ${}^{12}$C, or metastable states in cold atomic systems. While the inflection-point method discussed here seems to robustly capture the real part $E_R$ of the overall resonance position, more formal developments are necessary to extract resonance width from the details of the spectrum. There is some interesting recent work in this direction [48]. Overall, there are many exciting opportunities for future research.

## Footnotes

1 The DVR construction along Eqs. (32) and (35) is however much more general and needs not start from a plane-wave basis. For example, Refs. [59, 60] use a DVR basis built out of harmonic oscillator states in momentum space.

2 Note that for this identification it is important that the points ${-}L/2$ and *L*/2 are identified through the periodic boundary condition because otherwise the weight $w_{{-}n/2}$ would be incorrect.
